# Effects of Oxygen-Containing Functional Groups on the Electrochemical Performance of Activated Carbon for EDLCs

**DOI:** 10.3390/nano13020262

**Published:** 2023-01-07

**Authors:** Ju-Hwan Kim, Seok-Hwi Kim, Byung-Joo Kim, Hye-Min Lee

**Affiliations:** 1 Research & Development Division, Korea Carbon Industry Promotion Agency, Jeonju 54853, Republic of Korea; 2 Center for Bio-Resource Recycling, Institute for Advanced Engineering, Yongin 11780, Republic of Korea; 3 Department of Nano & Advanced Materials Engineering, Jeonju University, Jeonju 55069, Republic of Korea

**Keywords:** oxygen functional groups, activated carbon, electric double-layer capacitors, EDLCs

## Abstract

Activated carbon (AC) is used in commercial electric double-layer capacitors (EDLC) as electrode active material owing to its favorable properties. However, oxygen functional groups (OFGs) present in AC reduce the lifespan of EDLCs. Thus, we investigated the correlation between the OFGs in AC and their electrochemical characteristics. Samples were prepared by heat-treating commercial AC at 300 °C–900 °C for 1 h under two gas atmospheres (N_2_ and 4% H_2_/N_2_ mixed gas). The textural properties were studied, and the reduction characteristics of AC under Ar and H_2_/Ar mixed gas atmospheres were investigated. Additionally, changes in the OFGs with respect to the heat-treatment conditions were examined via X-ray photoelectron spectroscopy. The specific surface areas of AC-N and AC-H were 2220–2040 and 2220–2090 m^2^/g, respectively. Importantly, the samples treated in hydrogen gas exhibited a higher yield than those treated in nitrogen while maintaining their pore characteristics. Additionally, the electrochemical performance of the AC was significantly enhanced after the reduction process; the specific capacitance increased from 62.1 F/g to 81.6 F/g (at 0.1 A/g). Thus, heat treatment in hydrogen gas improves the electrochemical performance of EDLCs without destroying the pore characteristics of AC.

## 1. Introduction

Electric double-layer capacitors (EDLCs) are widely used as energy-storage devices in various high-power applications because of their high charge–discharge rates, long cycle lives, and high power densities [1,2,3,4]. Recently, EDLCs have been used in the fields of renewable energy and eco-friendly vehicles as auxiliary power sources for lithium-ion batteries (LIBs) with low output densities [4,5,6,7]. Furthermore, EDLCs can complement LIBs to achieve high power outputs in a short time [8].

In theory, the energy density of an EDLC is determined as 1/2 CV^2^, and higher energy densities can be achieved by using higher allowable voltages instead of increasing the specific capacitance [1,4]. Therefore, commercial EDLCs use organic electrolytes—which can run at a high voltage—and activated carbon (AC) as the electrode active material, because of its advantages such as a low price, simple manufacturing process, large specific surface area, and excellent pore properties.

AC is manufactured via a physical or chemical activation method that uses an oxidation reaction, in which abundant oxygen functional groups (OFGs) are formed at the grain edges [9,10,11]. The OFGs affect not only the potential of zero charges and electrolyte wettability of the pores [12] but also the capacitance [13,14] and inner resistance of the EDLC [15]. In particular, OFGs reduce the lifespan of the EDLC by causing side reactions with organic electrolytes during the repeated charging/discharging of the EDLC [16]. Therefore, many studies have been conducted to improve the electrochemical properties and lifespan of EDLCs by removing OFGs from AC [17,18,19]. Li et al. reported that the specific capacitance of AC was improved by approximately 10% as the ratio of OFG decreased from 7.42% to 2.62% [18]. Yang et al. reported that, as the OFG content of AC decreased from 2.4 mmol/g to 1.3 mmol/g, the specific capacitance retention—as indicated by the increase in current density (0.5–10.0 A/g)—improved from 38% to 59% [19]. Many studies have focused only on the correlation between OFGs and electrochemical properties, whereas few in-depth studies have been conducted on the effect of changes in the pore structure occurring during OFG removal on the electrochemical properties.

In this study, the effects of OFGs in AC on the electrochemical properties of EDLCs were analyzed by selectively controlling the OFGs through modification of the temperature (in the range of 300–900 °C) and composition of the gas atmosphere (N_2_ or 4% H_2_/N_2_ gas). The control mechanism for OFGs in AC was set up through temperature-programmed reduction (TPR) and surface chemistry analyses. OFG-controlled AC was prepared as a coin-cell-type EDLC using a 1 M (C_2_H_5_)_4_NBF_4_/propylene carbonate (PC) electrolyte. A control method for the OFGs was developed by analyzing the electrochemical characteristics with the aim of improving those of the EDLC without affecting the pore properties or electrical conductivity of the AC.

## 2. Materials and Methods

### 2.1. Activation

Coconut-shell-based AC (*S*_BET_: 1800 m^2^/g, Haycarb PLC, Badalgama, Sri Lanka) was used in the experiment. In accordance with our previous study, the coconut-shell-based AC was additionally activated by steam activation to obtain a high specific capacitance [20]. The AC was then placed in alumina boats (3 g each), which were inserted into a self-manufactured cylindrical tubular furnace (SUS tube: 100 × 1000 mm^2^), and heated to an activation temperature of 900 °C under a nitrogen atmosphere (N_2_, 99.999%, 300 cc/min) at a rate of 10 °C/min. The gas flow was then converted into water vapor at a rate of 0.5 mL/min and maintained for 40 min. Finally, the AC was naturally cooled to room temperature under the N_2_ gas atmosphere.

### 2.2. Characterization

#### 2.2.1. Reduction

After being placed in alumina boats (1 g each), the additionally activated AC was heat-treated in a custom-made high-temperature activation furnace (quartz tube: 130 × 1300 mm^2^) to control the OFGs formed during the activation process. Heat treatment was conducted for 1 h under an N_2_ gas or 4% H_2_/N_2_ gas atmosphere at 300 °C, 600 °C, and 900 °C at a rate of 10 °C/min, followed by natural cooling. The AC samples heat-treated under different gas atmospheres were named based on the following format: AC–atmosphere gas (N_2_ or 4% H_2_/N_2_)–reduction temperature; for example, AC-N-3/6/9 or AC-H-3/6/9.

#### 2.2.2. Surface Chemistry

The changes in the OFGs formed in AC according to the heat-treatment conditions were examined by X-ray photoelectron spectroscopy (XPS, PHI 5000, Versa Probe II, Japan). The X-ray anode was operated at >5 W, and the high voltage was maintained at 5.0 kV. The energy resolution was fixed at 0.50 eV. The pressure of the XPS analysis is on the level of 1.3 × 10^−6^ Pa. The peak in the O1s spectra were curve-fitted into four peaks: O1 (531.3 eV); O2 (532.3 eV); O3 (533.3 eV); and O4 (534.7 eV) with a Shirley baseline and Gauss–Lorentz function [21,22,23,24]. The center value and FWHM of four peaks (O1–O4 peak) were fixed during XPS fitting.

#### 2.2.3. OFG Control Mechanism

The control mechanism for the OFGs in AC was investigated using a TPR analyzer (BELCAT II, BEL Japan, Tokyo, Japan). First, 0.05 g of AC was placed in a U-shaped quartz tube and heated to 1000 °C at a rate of 1 °C/min under an Ar (99.999%, 50 mL/min) or hydrogen (4% H_2_/Ar, 50 mL/min) atmosphere. H_2_O, CO_2_, and CO generated during this process were detected using a mass spectrometer (BELMass, BEL Japan, Japan).

#### 2.2.4. Analysis of Pore Characteristics

The changes in the pore characteristics of AC according to the reducing conditions were measured using an isothermal gas adsorption analyzer (BELSORP-max, BEL Japan, Japan) after the AC was dried at 573 K for >12 h while the residual pressure was maintained below 0.133 Pa. The specific surface area was calculated from the isothermal adsorption curve using the Brunauer–Emmett–Teller (BET) equation [25], the micropore volume was calculated using the Dubinin–Radushkevich (DR) equation [26], and the mesopore volume was calculated by subtracting the micropore volume from the total pore volume. Pore size distribution (PSD) was calculated using non-localized density functional theory (NLDFT) [27].

### 2.3. Cell Production

The EDLC electrode was prepared by mixing the active material, conductive agent, and binder at a ratio of 84:7:9 wt.%, and the electrochemical properties of AC were examined along with changes in the OFGs. Carbon black (Super P, Timcal Ltd., Bodio, Switzerland) was used as the conductive agent. For the binder, carboxymethylcellulose (Dai-Ichi Kogyo Seiyaku Co., Ltd., Kyoto, Japan), styrene–butadiene rubber (BM400B, Zeon, Japan), and polytetrafluoroethylene (9002-84-0, Sigma-Aldrich, St. Louis, MO, USA) were used. The mixed slurry was coated on aluminum foil with a thickness of 0.152 mm using a laboratory-scale doctor blade coater. The coated electrode was dried overnight in a vacuum oven at 100 °C and then punched to a diameter of 12 mm. For the EDLC, the punched electrode, a 1 M (C_2_H_5_)_4_NBF_4_/PC organic solution (as an electrolyte), and a cellulose-based separator (NKK, Kanagawa, Japan) were assembled according to the CR2032 standard.

### 2.4. Electrochemical Tests

Cyclic voltammetry (CV) and impedance measurements of the EDLC were performed using a VSP electrochemical workstation (Bio-Logic Science Instruments, Grenoble, France), and galvanostatic charge–discharge (GCD) tests were conducted using a MACCOR 4300 battery tester (Maccor Inc., Tulsa, OK, USA). CV was performed at scan rates of 5 and 400 mV/s within the voltage range of 0.1–2.5 V, and impedance was measured within the frequency range of 10–300 mHz. GCD was measured with current densities of 0.1 and 10.0 A/g at voltages between 0.1 and 2.5 V. All electrochemical tests were conducted at room temperature (25 °C). To ensure the reliability of the results, each test was performed 10 times, and data from the 10th time were used. The specific capacitance (capacitance per unit electrode weight) of the EDLC was calculated using Equation (1) and the discharge curve of the GCD as well as the electrode weight.

(1) $$C_{g}=\frac{i\Delta t}{m\Delta v}$$

Here, *i* represents the discharge current (A), Δ*t* represents the discharge time (s), *m* represents the mass of the electrode, and Δ*v* represents the voltage (V).

## 3. Results and Discussion

### 3.1. TPR Analysis

**Figure 1:**
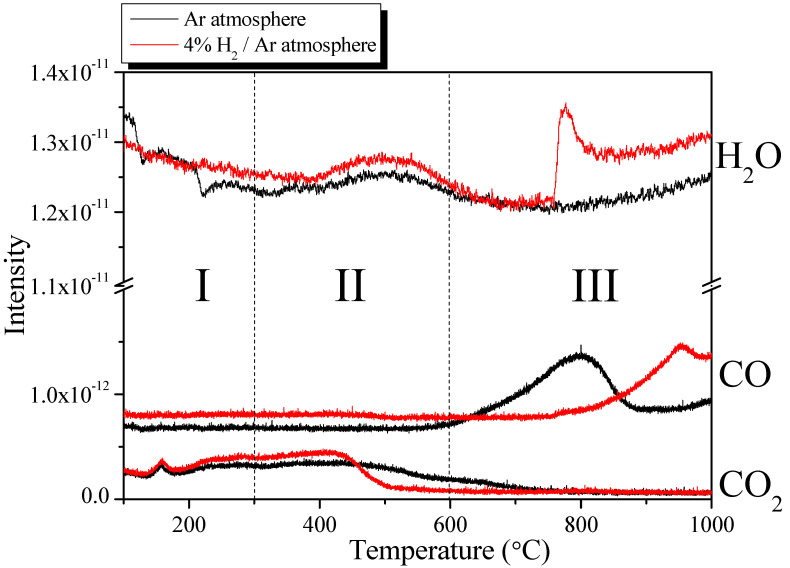
TPR spectra of the AC heated at 1 °C/min under different atmospheres.

TPR is used to identify oxidation and reduction characteristics of catalysts or solid samples by analyzing the changes in the concentrations of gases accompanying a linear increase in temperature. In this study, TPR was employed to investigate the differences in the control mechanism for the OFGs in AC under two types of gas atmospheres and at different temperatures. TPR was conducted under two gas atmospheres (Ar and 4% H_2_/Ar), and the peak intensities of the detected H_2_O, CO, and CO_2_ were recorded. Figure 1 shows the TPR spectra of the AC, while Table 1 presents the peak area value of the TPR spectra. The OFG control mechanism was divided into three stages according to the changes in the TPR spectra.

In general, the OFGs in AC are thermally decomposed into CO or CO_2_ under a nitrogen atmosphere [21,22,23,24,28,29]. However, under a hydrogen atmosphere, OFGs are reduced to H_2_O by combining with hydrogen or decomposed at a lower temperature than that under a nitrogen atmosphere because the required activation energy is lower [23,28]. Thus, the hydrogen atmosphere reduces the OFGs to H_2_O or decomposes them at a lower temperature, which are considered to be the cause of the different behaviors of the TPR spectra shown in Figure 1.

First, in Stage 1 (*T* < 300 °C), the H_2_O signal continuously decreased as the temperature increased. The OFGs adsorbed water in the atmosphere through hydrogen bonding. Although the AC was sufficiently dried at 100 °C for >24 h before the TPR analysis, residual H_2_O was apparently removed during the increase in temperature. In addition, at ≥200 °C, the H_2_O signal under a hydrogen atmosphere exhibited a higher intensity than that under the nitrogen atmosphere, indicating that the OFGs were reduced to H_2_O.

The CO signal exhibited a constant intensity regardless of the increase in temperature and a higher intensity under hydrogen atmosphere than that under nitrogen atmosphere. Conversely, the CO_2_ signal continuously increased as the temperature increased under all atmospheres. In particular, at ≥200 °C, the intensity of the CO_2_ signal in hydrogen was higher than that in nitrogen. This outcome indicates that, in Stage 1 (*T* < 300 °C), the OFGs were mostly decomposed into CO_2_ under nitrogen atmosphere, whereas they were removed under hydrogen atmosphere; not only through decomposition into CO_2_, but also thermal decomposition or reduction into CO or H_2_O.

In Stage 2 (300 °C < *T* < 600 °C), the H_2_O signal increased to 500 °C and then decreased under both gas atmospheres, and higher intensity was observed under the hydrogen atmosphere. According to Mudedla et al., carboxylic acid decomposes into CO_2_ or is converted into anhydride while generating H_2_O through hydrogen bonding with adjacent carboxylic acids [29]. Therefore, the H_2_O observed in Stage 2 was attributed to the conversion of carboxylic acid into anhydride, which formed H_2_O. Additionally, as in Stage 1, the OFGs were reduced to H_2_O in hydrogen, which supposedly induced a higher intensity than nitrogen.

Similar to that in Stage 1, the observed CO signal had a constant intensity under a hydrogen atmosphere. By contrast, the CO signal intensity under a nitrogen atmosphere was constant up to 500 °C and increased slightly thereafter. The CO_2_ signal intensity under the inert atmosphere was constant up to 500 °C and then decreased. The CO_2_ signal intensity under a hydrogen atmosphere increased from 300 °C to 450 °C and then decreased significantly until it was no longer visible beyond 500 °C. This finding indicated that, while most of the OFGs under the inert atmosphere were decomposed into CO_2_, they were decomposed into CO rather than CO_2_ at ≥500 °C. Similarly, the OFGs under a hydrogen atmosphere were decomposed into CO_2_ or CO but did not generate CO_2_ above 500 °C because of their rapid decomposition. 

Finally, in Stage 3 (*T* > 600 °C), the H_2_O signal intensity decreased as the heat-treatment temperature increased and then increased starting from approximately 750 °C under all atmospheres. In particular, an abnormal increase in intensity of the H_2_O signal was observed in hydrogen at 750 °C. However, many studies have indicated that the OFGs decompose only into CO and CO_2_ at ≥700 °C. Therefore, the increase in H_2_O signal intensity under the inert atmosphere in Stage 3 was apparently due to the structural limitations of the TPR analysis equipment, in which the condensed water between the heater and detector was evaporated by high temperatures. The observed H_2_O signal under a hydrogen atmosphere was attributed to the decomposition of OFGs into H_2_O.

The intensity of the CO signal under the inert atmosphere increased sharply up to 800 °C, decreased thereafter, and then increased again from 900 °C. By contrast, the intensity of the CO signal under a hydrogen atmosphere increased from 800 °C. The CO_2_ signal under the inert atmosphere continuously decreased and was not observed above approximately 800 °C, whereas that under the hydrogen atmosphere was not observed in Stage 3.

The results for Stage 3 confirmed that as the temperature increased, the OFGs under the inert atmosphere were decomposed in the order of CO_2_, H_2_O, and CO. Under a hydrogen atmosphere, the decomposition of OFGs was first observed at a lower temperature than under the inert atmosphere, and other decomposition mechanisms induced the decrease in CO generation and increase in H_2_O generation.

In Table 1, the area values of the CO_2_, CO, and H_2_O peaks changed similarly to the intensity change in the TPR spectra. In particular, the total H_2_O peak area value showed greater difference depending on the gas atmosphere than the CO and CO_2_ peak area values (Ar atmosphere: 35.83 × 10^−11^, 4% H_2_/Ar atmosphere: 92.55 × 10^−11^). The results indicated that pyrolysis under H_2_/N_2_ atmosphere induced the removal process at a lower temperature compared with that under the inert atmosphere and removed OFGs as H_2_O rather than CO or CO_2_.

### 3.2. Surface Chemistry

In general, the OFGs in AC include carboxylic acid, anhydride, phenol, ether, carbonyl groups, and quinone, and decomposition starts in the order of carboxylic acid (373–673 K) < anhydride (623–900 K) < phenol (873–973 K) < ether, carbonyl, and quinone (973–1253 K) owing to the differences in the level of activation energy required for OFG removal [21,24,30]. Therefore, for selectively controlling the OFGs, the control process in this experiment was conducted for 1 h at 300 °C, 600 °C, and 900 °C, under nitrogen or 4% hydrogen atmosphere.

**Figure 2:**
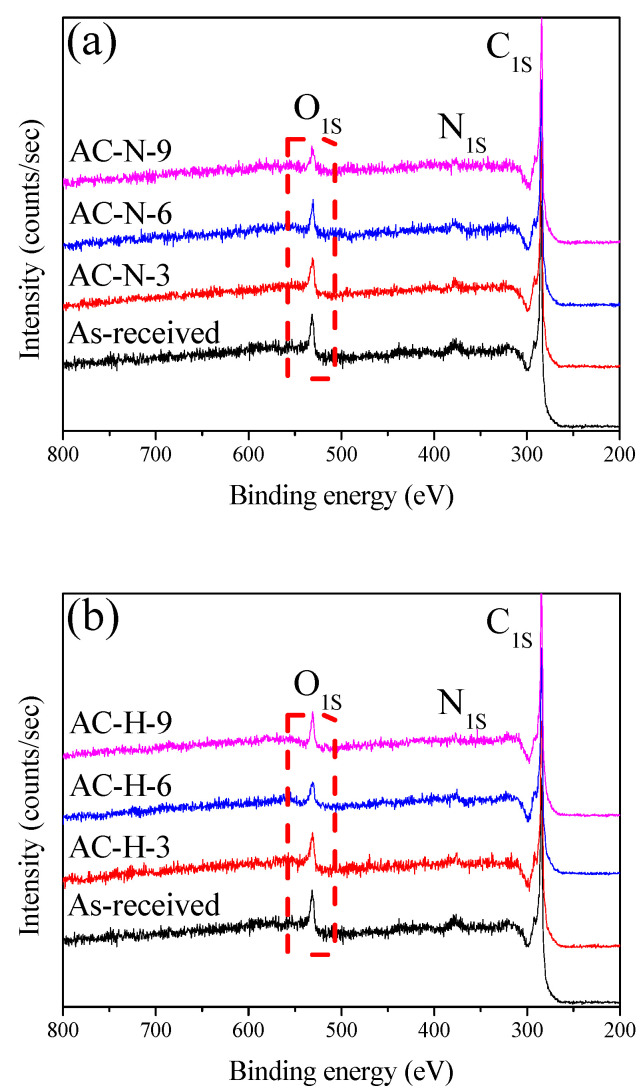
XPS survey spectra of AC heat-treated under different conditions; (**a**) AC-N, (**b**) AC-H.

XPS is a nondestructive (or weak) surface technique in which the electron binding energies of atoms present on the sample surface are used to determine the elemental composition and chemical states of the surface. Figure 2 shows the survey spectra of the AC at each stage, with the OFGs being controlled.

**Figure 3:**
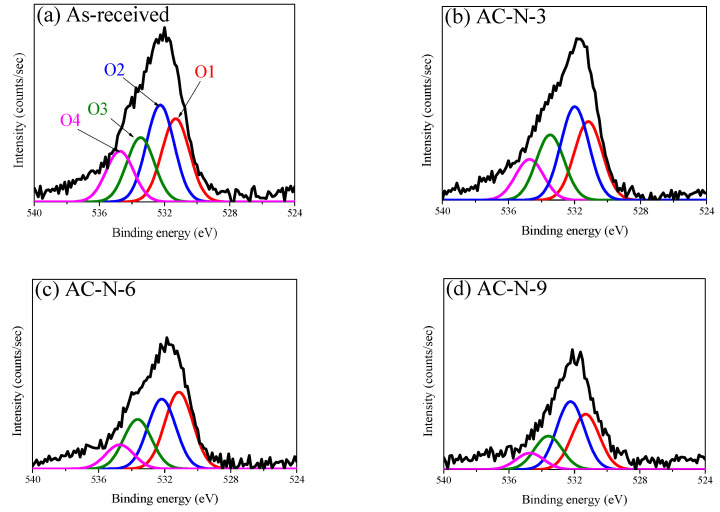
High-resolution O_1S_ spectra of AC-N; (**a**) As-received, (**b**) AC-N-3, (**c**) AC-N-6, (**d**) AC-N-9.

**Figure 4:**
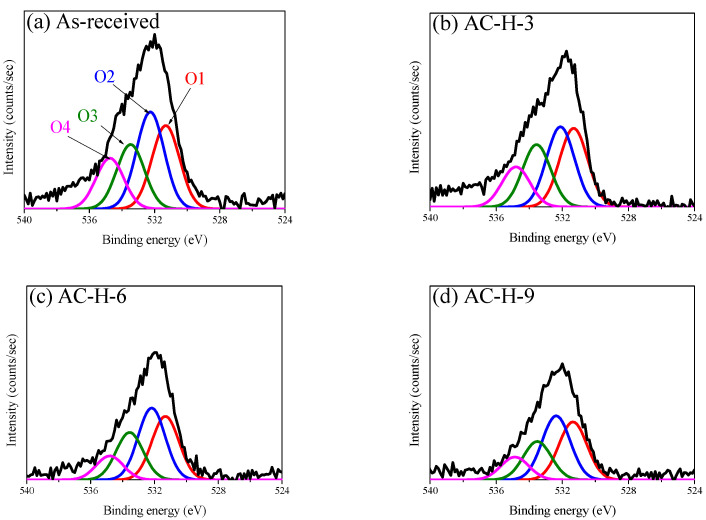
High-resolution O_1S_ spectra of AC-H; (**a**) As-received, (**b**) AC-H-3, (**c**) AC-H-6, (**d**) AC-H-9.

**Figure 5:**
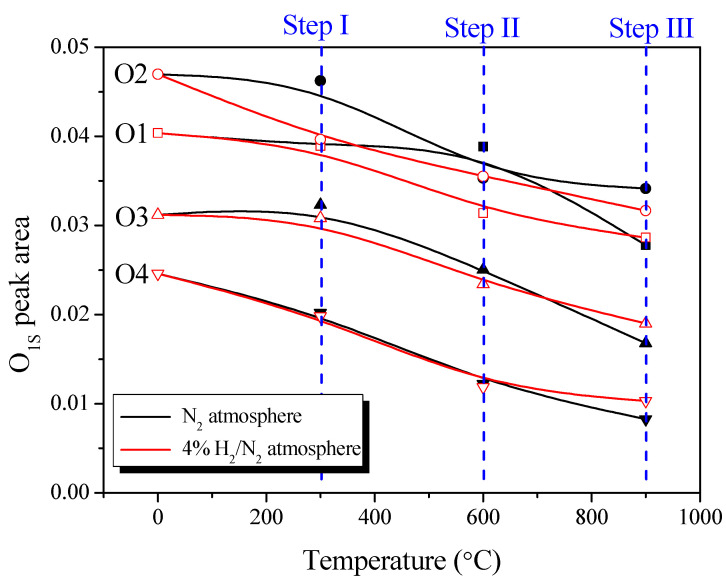
The results of deconvoluted high-resolution O_1S_ spectrum of AC-N, AC-H.

Substantial changes in OFGs due to pyrolysis or reduction can be identified by examining the O_1*s*_ core region. As shown in Figure 3 and Figure 4, to determine the OFGs on the surface of the AC, the O_1*s*_ spectra were curve-fitted into four individual peaks: C=O in carbonyl and quinone (O1, 531.3 eV); C-OH in phenol, C-O in ether, and C=O in anhydride (O2, 532.3 eV); C-O-C in anhydride (O3, 533.3 eV); and carboxylic acid (O4, 534.7 eV) [21,22,23,24]. Figure 5 also shows the changes in the areas of the fitted O_1*s*_ peaks. The O_1*s*_ fitting results indicated that the reduction in OFG content differed significantly between the two gas atmospheres (N_2_ and 4% H_2_/N_2_).

The O1 peak (C=O in carbonyl, quinone) under a nitrogen atmosphere was reduced only in Stage 3; by contrast, the O1 peak under a hydrogen atmosphere continued to decrease from Stages 2 to 3 and was lower than that under nitrogen at 900 °C. Carbonyl and quinone decompose to CO above 973 K (700 °C) [21,22]. Accordingly, the O1 peak under the inert atmosphere was reduced only in Stage 3, and CO was observed from 600 °C, as shown in Figure 1. However, for the O1 peak under the hydrogen atmosphere, the O in the C=O bond was reduced with hydrogen to H_2_O at a temperature lower than the pyrolysis temperature. This explains the higher intensity of the H_2_O signal in Stages 2 and 3 under hydrogen atmosphere than that under a nitrogen atmosphere, as shown in Figure 5. 

The O2 peak under the inert atmosphere (C=O in anhydride (decomposition start temperature: 623 K), C-OH in phenol (decomposition start temperature: 873 K), C-O in ether (decomposition start temperature: 973 K)] decreased from Stage 2 to Stage 3. Anhydride decomposes into CO or CO_2_ under an inert atmosphere, whereas phenol and ether decompose into CO [21,24]. Therefore, in Stage 2, the O2 peak under the inert atmosphere was reduced by the decomposition of anhydride and phenol; during this process, CO_2_ and CO were generated, as confirmed by the signals shown in Figure 1. The increase in CO near 600 °C under the inert atmosphere, as shown in Figure 1, is attributed to the decomposition of phenol. Thereafter, in Stage 3, the O2 peak under the inert atmosphere decreased owing to the decomposition of ether at a high decomposition temperature, which was considered to be the reason for the high intensity of the CO signal in Stage 3.

The O2 peak in the hydrogen atmosphere decreased significantly from Stage 1 and continued to decrease thereafter. As shown in Figure 1, the intensities of the H_2_O and CO signals in Stages 1 and 2 were higher under a hydrogen atmosphere than that under a nitrogen atmosphere, and the CO_2_ signal intensity remained high up to approximately 450 °C before rapidly decreasing. Subsequently, in Stage 3, the CO_2_ signal was not observed in hydrogen, while the H_2_O and CO signals increased in intensity from approximately 800 °C. This finding indicated that, under a hydrogen atmosphere, OFGs were thermally decomposed into H_2_O, CO_2_, or CO at a rapid rate from a lower temperature than that under an inert atmosphere. In particular, Figure 1 shows the reduction in CO_2_ signal intensity near 450 °C, which was attributed to the decomposition of OFGs at a lower temperature.

In contrast to the O1 and O2 peaks, the O3 and O4 peaks were similar between the different gas atmospheres, indicating that O3 and O4 were more significantly affected by the decomposition temperature than the gas atmosphere.

The O3 peak (C-O-C in anhydride, decomposition start temperature: 623 K) did not change significantly in Stage 1 regardless of the gas atmosphere, but decreased from Stage 2 to Stage 3. These deviating results were attributed to the influence of the thermal decomposition temperature of C-O-C; in particular, the O3 peak in the hydrogen atmosphere decreased slightly in Stage 1 ahead of that in the inert atmosphere. Similar to the change in the O2 peak (C=O in anhydride) that was previously presented, the results indicated that the C-O-C in anhydride was decomposed accompanying the removal of anhydride, which increased the intensities of the CO_2_ and CO signals, as shown in Figure 1.

The O4 peak (carboxylic acid, decomposition start temperature: 373 K) decreased under all atmospheres at similar rates as the temperature increased. Thus, although its pyrolysis started at a temperature lower than those of other OFGs, carboxylic acid was not completely removed and continued to decrease until Stage 3.

### 3.3. Adsorption Isotherms and Textural Properties

**Figure 6:**
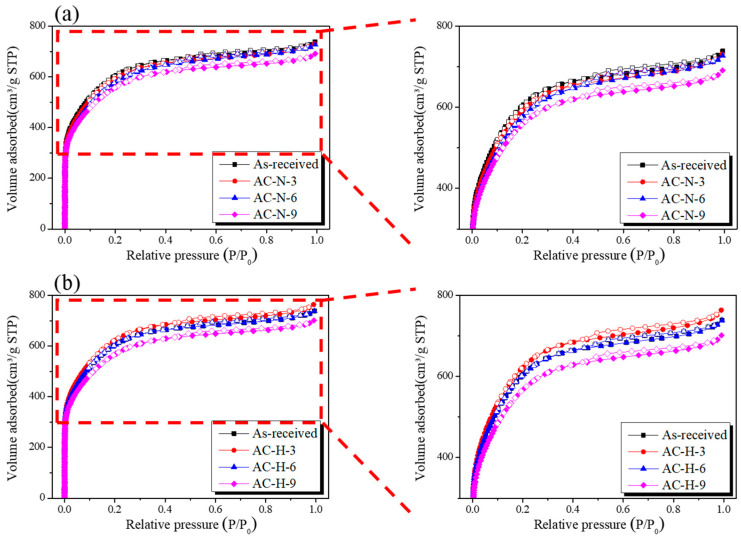
N_2_/77 K adsorption isotherms of AC heat-treated under different gas atmospheres: (**a**) N_2_ and (**b**) H_2_/N_2_ mixed gas.

The N_2_/77 K isotherm adsorption–desorption curve, which is the most effective method for analyzing the pore characteristics of AC, was used to investigate the changes in the pore characteristics of the AC according to the heat-treatment conditions. Figure 6 shows the N_2_/77 K adsorption–desorption curves of AC-N and AC-H. The N_2_/77 K adsorption–desorption curves are divided into eight types according to the International Union of Pure and Applied Chemistry (IUPAC) standards [31], thereby allowing the pore characteristics of the adsorbent to be analyzed according to the shape of the curve. As shown in Figure 6, all of the isothermal adsorption curves of the AC had the form of Type I(b) according to the IUPAC standards [31], indicating that micropores and mesopores developed simultaneously in all AC samples.

As shown in Figure 6a, as the pyrolysis temperature increased, the N_2_ adsorption of AC-N remained unchanged up to 600 °C and then decreased at 900 °C. By contrast, Figure 6b shows that the N_2_ adsorption of AC-H increased slightly as the heat-treatment temperature increased up to 600 °C and then decreased at 900 °C, similar to AC-N. As indicated by Figure 1, the OFGs of AC were removed with a higher efficiency under a hydrogen atmosphere than under the inert atmosphere owing to the generation of H_2_O, CO, and CO_2_ during Stages 1 and 2. Therefore, N_2_ adsorption increased slightly for AC-H owing to the etching of the crystal structure during the removal of OFGs into CO and CO_2_. By contrast, for AC-N-9 and AC-H-9, N_2_ adsorption decreased owing to the etching of grain edges, as abundant OFGs were removed into CO.

The shapes of hysteresis loops have been correlated to specific pore morphologies [31]. H4-type hysteresis based on the IUPAC standards was observed in all AC isothermal adsorption curves [31], and no significant change was observed in the area of the hysteresis loop even when the reduction temperature was increased. This finding indicated that all ACs had similar slit-shaped pores and that the removal of OFGs did not affect the pore shapes of the AC.

Table 2 presents the textural properties of ACs heat-treated under different conditions. As the heat-treatment temperature increased, the heat-treatment yields of AC-N and AC-H decreased to 99.4%–69.0% and 99.0%–88.4%, respectively. AC-N-9 showed the lowest heat-treatment yield (69.0%), indicating that the heat-treatment under a hydrogen atmosphere removed OFGs as H_2_O instead of CO or CO_2_ at a relatively low temperature, resulting in a higher yield compared to that obtained under the inert atmosphere.

The specific surface area and total pore volume of AC-N decreased to 2200–2040 m^2^/g and 1.14–1.07 cm^3^/g, respectively, while AC-N-9 exhibited the most significant degradation in the pore properties. The specific surface area and total pore volume of AC-H increased slightly up to 300 °C and then decreased to 2200–2090 m^2^/g and 1.16–1.08 cm^3^/g, respectively, as the temperature further increased. AC-H-9 exhibited the most significant degradation in pore properties among the AC samples heat-treated under the hydrogen atmosphere.

The mesopore ratios of AC-N and AC-H increased to 36.0–40.2% and 36.0–38.0%, respectively, as the pyrolysis temperature increased to 600 °C and then maintained their respective levels. The mesopore ratio of AC-N was higher than that of AC-H. Because OFGs are mainly formed at the edge of the carbon crystal structure, the mesopore volumes of AC-N and AC-H increased, presumably because of etching of the grain edge caused by the removal of the OFGs. In particular, in AC-N, most of the OFGs were removed as CO or CO_2_; therefore, a larger change in the mesopore ratio was observed in AC-N compared to that in AC-H.

**Figure 7:**
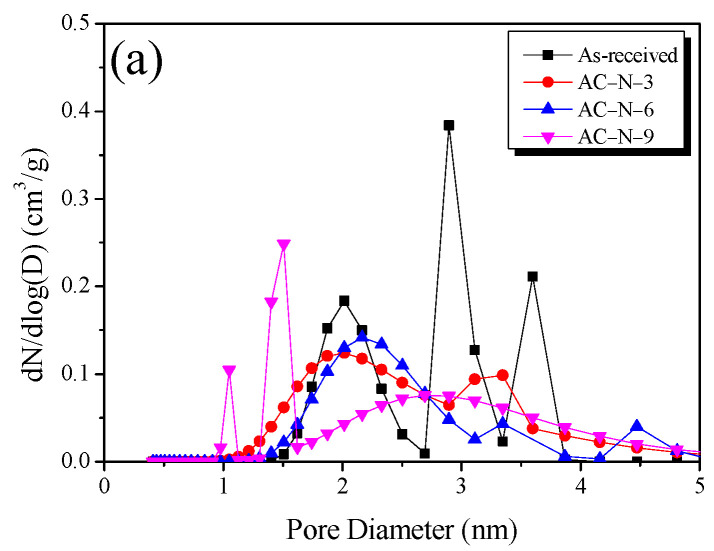

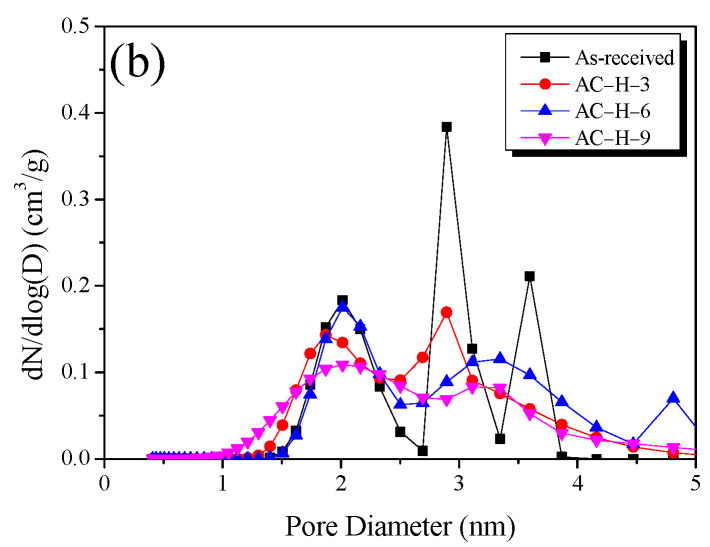
Pore size distribution of AC heat-treated under different atmospheres based on the NLDFT method: (**a**) N_2_; (**b**) H_2_/N_2_ mixed gas.

PSD curves based on NLDFT are useful for analyzing the pore distribution of AC. Figure 7a,b shows the PSD of AC-N and AC-H, respectively. AC-N and AC-H exhibited different change behaviors of the PSD according to the OFG removal process. As shown in Figure 1, the OFGs are decomposed by different mechanisms depending on the gas atmosphere. As the temperature increased, the OFGs at the grain edges were removed as CO and CO_2_ under the inert atmosphere, resulting in the oxidation of grain edges and increased pore diameter via pore drilling. Accordingly, the pore diameter and mesopore volume increased even though only heat-treatment was performed under inert and hydrogen atmospheres. For the AC-N heat-treated under the inert atmosphere, the pore diameter and mesopore volume increased significantly as the heat-treatment temperature increased. In particular, the substantial generation of CO at 800 °C shown in Figure 1 significantly promoted the etching reaction at the grain edges, thereby reducing the volume of pores with diameters of <2 nm in the PSD of AC-N-9. However, in AC-N-9, the formation of micropores (with diameters of <1.5 nm) was observed together with >30% burn-off, as well as significant etching reaction at the grain edges, indicating that new micropores were formed inside the pores.

Under a hydrogen atmosphere, OFG removal occurred at a lower temperature than under the inert atmosphere, and a smaller increase in the pore diameter was observed even at the same pyrolysis temperature because a large portion of OFGs was reduced to H_2_O. The results indicated that the OFG removal process under the inert atmosphere caused deformation in the AC pore structure through the oxidation of grain edges; by contrast, OFG removal caused relatively minimal deformation and selectively removed specific OFGs under a hydrogen atmosphere.

### 3.4. Electrochemical Performance

**Figure 8:**
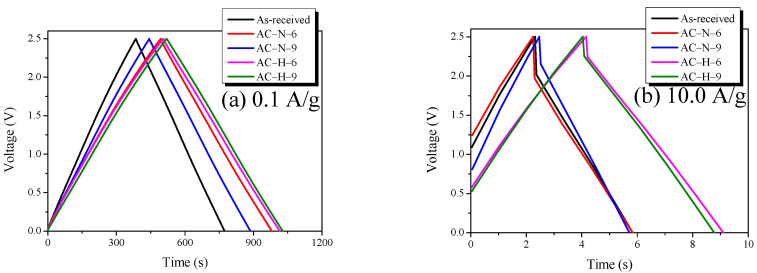
Galvanostatic charge–discharge curves of AC heat-treated under different conditions: (**a**) 0.1 A/g; (**b**) 10.0 A/g.

GCD curves are widely used for measuring the electrochemical performance of EDLCs. The GCD curve, in which the charge and discharge curves are symmetric, indicates the typical capacitive behavior of EDLCs. The voltage drop (*IR* drop) observed when the discharge line starts indicates the ohmic resistance of the system and the inner resistance of ionic diffusion in nanopores. Figure 8 shows the GCD curve of AC. Table 3 presents the specific capacitance of the ACs.

As shown in Figure 8a, all AC samples had symmetric charge and discharge curves, and small *IR* drops were observed. These results indicated that all AC samples had low inner resistances owing to the typical EDLC charging/discharging behavior based on the non-Faradic process, as well as excellent pore characteristics, with specific surface areas of >2000 m^2^/g. By contrast, as shown in Figure 8b, the ohmic resistance of the system increased as the current density was increased to 10.0 A/g. Thus, the *IR* drop of the GCD curve was clearly observed. The largest *IR* drops were observed for the as-received and AC-N-6 samples, followed by AC-N-9, AC-H-6, and AC-H-9. This trend indicated that a higher thermal decomposition temperature corresponded to a lower OFG content of AC, as well as a smaller IR drop. Thermal decomposition under the hydrogen atmosphere had a more positive effect on the *IR* drop than that under the inert atmosphere.

While the specific surface areas of all AC samples were similar, the specific capacitance depended on the gas atmosphere during pyrolysis.

The specific capacitances of AC-N increased to the ranges of 62.1–77.8 and 52.0–57.4 F/g at the current densities of 0.1 and 10.0 A/g, respectively. By contrast, the specific capacitances of AC-H increased to the ranges of 62.1–81.6 and 52.0–79.2 F/g at the current densities of 0.1 and 10.0 A/g, respectively. Therefore, AC-H, which was pyrolyzed under a hydrogen atmosphere, exhibited the highest specific capacitance, followed by AC-N and the as-received sample, which were pyrolyzed in an inert atmosphere. Compared with the as-received sample, AC-H-6 showed the highest specific capacitance, which increased by >30% (62.1 F/g to 81.3 F/g) and >50% (52.0 F/g to 79.2 F/g) at current densities of 0.1 and 10.0 A/g, respectively. AC-N exhibited only approximately 10% improvement compared with the as-received sample regardless of current density, indicating that the electrochemical properties of AC treated under the hydrogen atmosphere were more positively affected by the pyrolysis method than those under the inert atmosphere.

**Figure 9:**
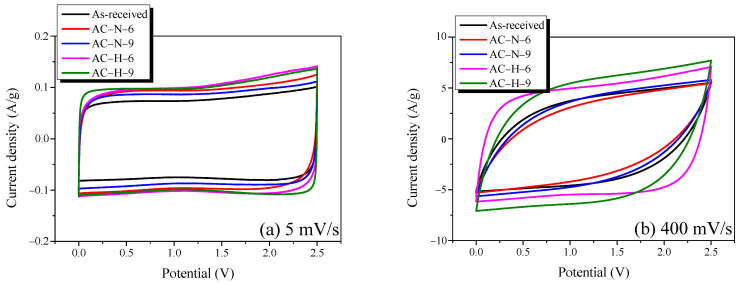
Cyclic voltammograms of AC heat-treated under different conditions: (**a**) 5 mV/s; (**b**) 400 mV/s.

Figure 9 shows the cyclic voltammograms of AC measured at scan rates of 5 and 400 mV/s. In Figure 9a, all CV curves of AC exhibit an ideal rectangle shape, and no Faradic redox reaction is observed. This result indicates that all AC samples possess excellent pore characteristics—and, thus, exhibit ideal EDLC charge–discharge behavior—at a scanning rate of 5 mV/s, and that the redox reaction attributed to OFGs does not occur. Meanwhile, in Figure 9b, all CV curves of AC show a leaf shape because the resistance increased with the scan speed.

The areas of the CV curves decreased in the order of AC-H > AC-N > as-received; this trend was in agreement with the previously described GCD results, regardless of the scanning speed. The OFGs were mainly formed at the grain edges of AC, reducing the electrical conductivity and negatively affecting the formation of an electrochemical double-layer for non-Faradic capacitance [32,33]. Thus, similar to the specific capacitance described in Figure 6, AC-H had the best electrochemical performance despite the similar specific surface areas of all AC samples because of the effect of OFGs under the gas atmosphere.

**Figure 10:**
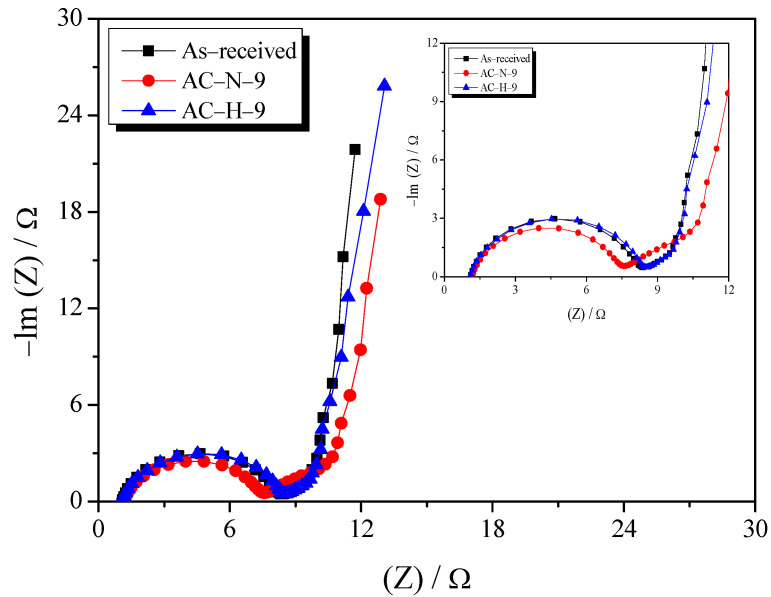
Nyquist plot of AC heat-treated under different conditions.

Electrochemical impedance spectroscopy is mainly used to analyze the impedance of EDLCs. Figure 10 shows the Nyquist plot of the AC obtained in the frequency range 10 mHz–300 kHz. Two representative samples were selected for comparing the effects of gas atmosphere on the pyrolysis process. The Nyquist plot consists of three elements: the bulk solution electrolyte resistance (*R*_S_), charge-transfer resistance (*R*_CT_), and Warburg impedance (*R*_W_) [34].

The first element of the Nyquist plot is the bulk-solution electrolyte resistance (*R*_S_), which can be easily identified using the x-intercept at high frequencies. The *R*_S_ depends on the type or concentration of the electrolyte used for the EDLC. Because the same electrolyte (1 M TEABF_4_/PC) was used in this study, similar *R*_S_ values were obtained (1.06–1.30 Ω).

The second element is the *R*_CT_, which is represented by a semicircle in the intermediate frequency range. *R*_CT_ is a composite resistance that accounts for various factors, such as the electrical conductivity of the electrode active material, interfacial resistance occurring at the interface between the active material and the electrolyte, and contact resistance between the electrode and current collector. The *R*_CT_ values of AC-N-9 and AC-H-9 were similar to those of the as-received sample (6.42–7.41 Ω). AC-N-9 and AC-H-9 were presumed to have a positive effect on *R*_CT_ through the reduced interfacial resistance and increased electrical conductivity, which were attributed to the removal of abundant OFGs, as well as a negative effect on the formation of the electric double layer owing to the increase in pore diameter during the OFG removal process. Similar *R*_CT_ values were observed for all samples, including the as-received sample, because of these complex factors. However, AC-N-9 had the smallest *R*_CT_ value because of the development of new micropores, as shown in Figure 7.

The last element of the Nyquist plot is the Warburg impedance (*R*_W_), which is represented by a straight line with a slope of 45° at low frequencies. *R*_W_ indicates the diffusion resistance of electrolyte ions. AC-H-9 and the as-received sample had similar *R*_W_ values, while AC-N-9 had a higher R_W_ value than the other ACs. The *R*_W_ of AC was the most significantly affected by the PSD of the AC [35]. In the previously presented results for the pore characteristics, AC-H-9 exhibited no significant change in PSD due to the removal of OFGs into H_2_O, whereas AC-N-0 exhibited a burn-off of ≥30%, as well as a change in PSD, due to an increase in the volume of mesopores (increase in the pore diameter) along with the removal of OFGs into CO. These findings indicated that AC-N-9 formed deeper pores compared with those of the as-received sample during the thermal stabilization process and, thus, had a higher *R*_W_ than the other ACs.

The OFG removal processes under all gas atmospheres yielded similar specific surface areas and minimal changes in *R*_S_ and *R*_CT_. Meanwhile, for OFG removal under the inert atmosphere, the PSD changed owing to the oxidation of the grain edges, expanding the pores inside, which increased *R*_W_. Thus, to improve the electrochemical performance of AC through OFG removal, a method for effectively removing OFGs while generating less burn-off is needed to avoid affecting the pore structure. To this end, heat-treatment in the hydrogen atmosphere was confirmed to be a suitable technique.

## 4. Conclusions

The effects of the OFG removal process for AC treated under gas atmospheres on the electrochemical properties of the fabricated EDLC were examined. The OFGs in AC were selectively controlled through heat-treatment in the temperature range of 300–900 °C under N_2_ or 4% H_2_/N_2_ atmospheres. By analyzing the H_2_O, CO, and CO_2_ generated during the removal process, we confirmed that different mechanisms contributed to the decomposition of OFGs. In particular, the results indicated that pyrolysis under the H_2_/N_2_ atmosphere induced the removal process at a lower temperature than that under the inert atmosphere, and removed OFGs as H_2_O rather than CO or CO_2_.

For AC-N and AC-H, the specific surface area decreased to 2220–2040 and 2220–2090 m^2^/g, respectively, as the heat-treatment temperature increased, and the pore diameter and mesopore volume increased owing to etching of the grain edges. For AC-N-9, new pores were formed inside the pore structure.

The results also confirmed that the electrochemical properties of AC-N and AC-H were affected by the pore and surface chemical properties of AC, which depended on the heat-treatment temperature. The electrochemical performance of AC-N-9 and AC-H-9 exhibited more significant improvements than that of the as-received sample with the removal of OFGs. The specific capacitance of AC-H increased by >30% compared with that of the as-received sample and increased by >50% at a high current density (10 A/g), not only increasing the energy density but also the output density. Thus, while the electrochemical properties of EDLCs can be improved by simply reducing the total content of OFGs, the results of this study confirmed the possibility of further improvement through the selective removal of only the OFGs that affect the conductivity of AC and interfacial resistance between organic electrolytes, thereby maintaining the pore characteristics of the AC.

In conclusion, to improve electrochemical performance through the removal of the OFGs of AC, a method for effectively removing OFGs while generating less burn-off is needed to avoid affecting the pore structure. This study confirmed the feasibility of heat-treatment under a hydrogen atmosphere.

## Figures and Tables

**Table 1:** Peak area values of the TPR spectra.

	CO_2_ (10^−11^)		CO (10^−11^)		H_2_O (10^−11^)	
	Ar	4% H_2_/Ar	Ar	4% H_2_/Ar	Ar	4% H_2_/Ar
Stage I	5.74	6.63	1.50	3.73	13.59	14.14
Stage II	8.89	8.43	2.13	5.31	12.98	17.52
Stage III	3.66	2.83	15.63	14.24	9.26	25.06
Total	18.29	17.89	19.26	23.28	35.83	92.55

**Table 2:** Textural properties of AC heat-treated under different conditions.

Sample Name	*S*_BET_^a^ (m^2^/g)	V_total_^b^ (cm^3^/g)	V_micro_^c^ (cm^3^/g)	V_meso_^d^ (cm^3^/g)	R_M_^e^ (%)	Y ^f^ (%)
As-received	2220	1.14	0.7	0.41	36	-
AC-N-3	2180	1.13	0.69	0.44	38.9	99.4
AC-N-6	2120	1.12	0.67	0.45	40.2	82.8
AC-N-9	2040	1.07	0.64	0.43	40.2	69
AC-H-3	2290	1.18	0.73	0.45	38.1	99
AC-H-6	2200	1.16	0.71	0.45	38.8	92.8
AC-H-9	2090	1.08	0.67	0.41	38	88.4

^a^*S*_BET_: Specific surface area; BET method $\frac{P}{v\left(P_{0}-P\right)}=\frac{1}{v_{m}c}+\frac{c-1}{v_{m}c}\frac{P}{P_{0}}$; ^b^*V*_Total_: Total pore volume; amount adsorbed *P*/*P*_0_ = 0.99; ^c^ *V*_Micro_: Micropore volume; DR method $ln(V_{\mathrm{ads}})=\ln(V_{\mathrm{DR}})-{\left(\frac{R_{\mathrm{gas}}T}{E_{\mathrm{DR}}\beta }\right)}^{2}\times {\left[\ln\left(\frac{P_{0}}{P}\right)\right]}^{2}$; ^d^ *V*_Meso_: Mesopore volume; *V*_Total_–*V*_Micro_; ^e^ R_M_: Mesopore volume ratio; $\frac{V_{\mathrm{Meso}}}{V_{\mathrm{Total}}}\times 100$; ^f^ Y: Heat-treatment yield; $\frac{Weight\;of\;reducted\;sample}{Weight\;of\;sample\;input}\times 100.$

**Table 3:** Specific capacitance under different current densities of heat-treated AC.

Sample Name	Specific Capacitance at 0.1 A/g (F/g)	Specific Capacitance at 10.0 A/g (F/g)
As-received	62.1	52.0
AC-N-6	77.8	57.4
AC-N-9	70.9	56.3
AC-H-6	81.3	79.2
AC-H-9	81.6	75.8

## Data Availability

The data presented in this study are available on request from the corresponding author.
